# Supplementary figures and images for: Identification and characterization of large-scale genomic rearrangements during wheat evolution
Source: PLoS One. 2020 Apr 14;15(4):e0231323. doi: 10.1371/journal.pone.0231323 (PMC7156093; doi:10.1371/journal.pone.0231323)

**A**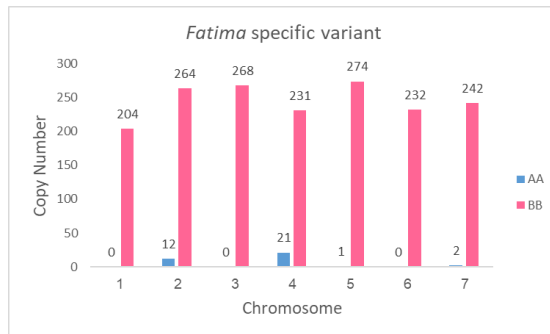**B**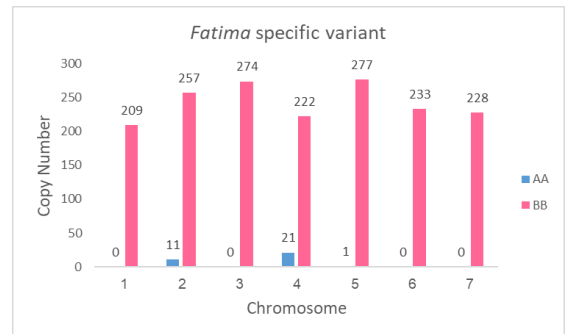

Supplement: S1 Fig — Distribution of Fatima specific variant in chromosomes 1–7 of sub-genome A (blue) and sub-genome B (pink) in wild emmer (A) and bread wheat (B). Unmapped Fatima insertions are not shown. (PDF) [file pone.0231323.s001.pdf]

**A**

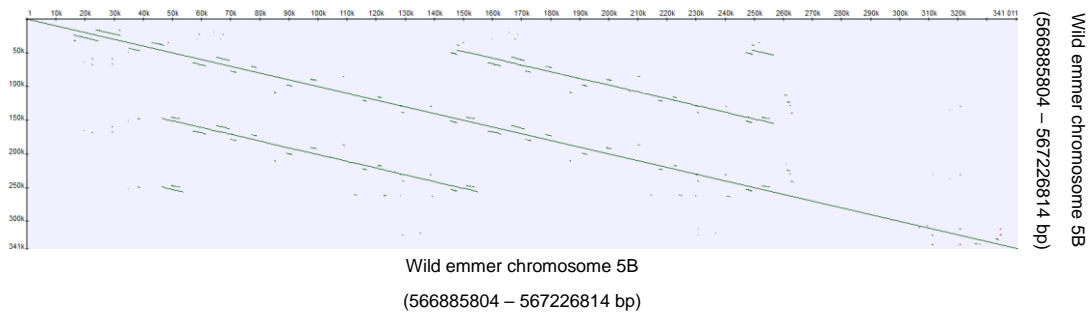

**B**

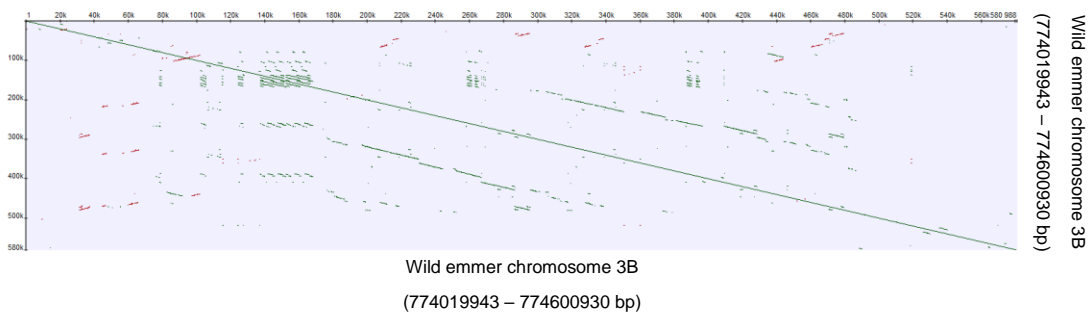

**C**

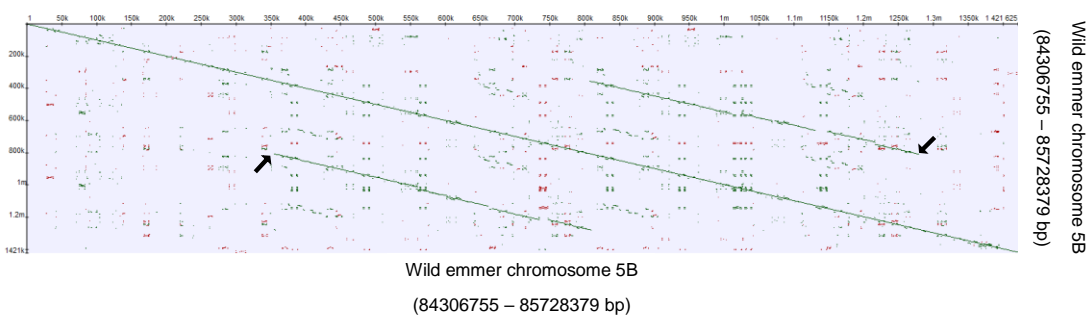

Supplement: S3 Fig — In 5B1 (A) and 3B1 (B) loci, the deleted sequences and the Indels borders were found to contain sequence duplications. (C) Recent sequence duplication in locus 5B6 identified in wild emmer relative to bread wheat. The parameters for the sequence alignments of the genomic loci against themselves were minimum repeat length of 100bp and 95% repeats identity. Green- direct repeats, red- inverted repeats. Indels break points\ borders are indicated by black arrows. The numbers in brackets refers to the coordinates of the selected sequences in the WEWSeq_v.1.0 assembly. (PDF) [file pone.0231323.s003.pdf]
