# Supplementary material for: Identification and characterization of large-scale genomic rearrangements during wheat evolution
Source: PLoS One. 2020 Apr 14;15(4):e0231323. doi: 10.1371/journal.pone.0231323 (PMC7156093; doi:10.1371/journal.pone.0231323)
Supplement: S2 Fig — Dot plot representations of genomic loci containing sequence variations identified between wild emmer (x axis) and bread wheat (y axis) in loci 5B1 (A) 3B1 (B) 5B2 (C) 5B3 (D) 5B4 (E) 3B2 (F) 3B3 (G) 3B4 (H) 3B5 (I) 5B5 (J) 5B6 (K) and a ~6.5 Mb inversion including the genomic locus 5B6 (L). The parameters for the sequence alignments were minimum repeat length of 100 bp and 95% repeats identity. Green- direct repeats, red- inverted repeats. Indels break points\ borders are indicated by black arrows. The numbers in brackets refer to the coordinates of the selected sequences in the WEWSeq_v.1.0 assembly (for wild emmer) and in the IWGSC assembly (for bread wheat). (PDF) [file pone.0231323.s002.pdf]

A

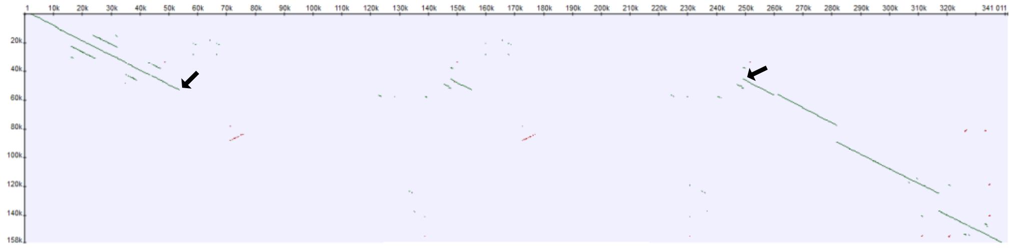

Bread wheat chromosome 5B
(561012086 – 561171024 bp)

Wild emmer chromosome 5B
(566885804 – 567226814 bp)

B

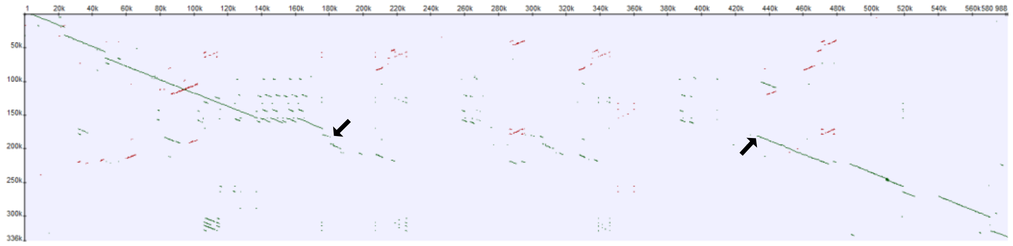

Bread wheat chromosome 3B
(760622966 – 760959530 bp)

Wild emmer chromosome 3B
(774019943 – 774600930 bp)

C

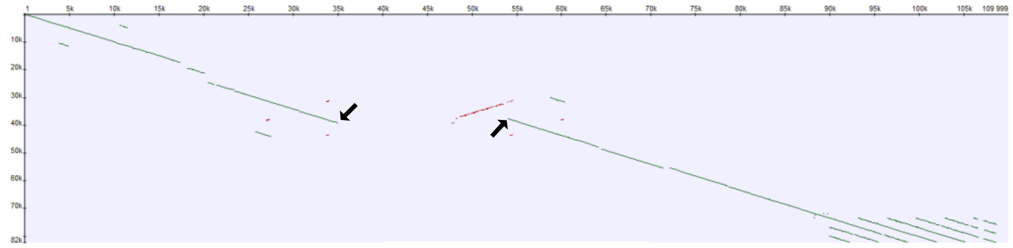

Bread wheat chromosome 5B
(511345836 – 511428124 bp)

Wild emmer chromosome 5B
(516667317 – 516777315 bp)

D

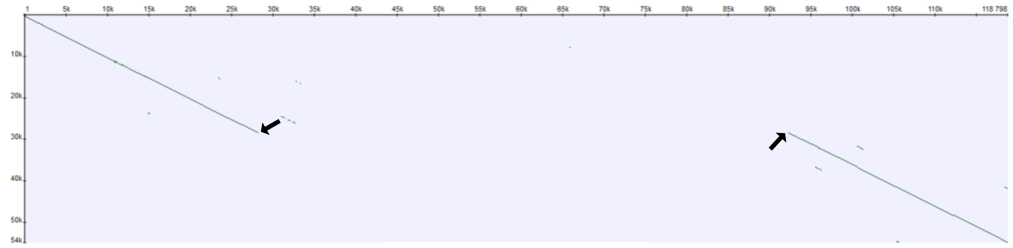

Bread wheat chromosome 5B
(349905881 – 349960770 bp)

Wild emmer chromosome 5B
(363459095 – 363577892 bp)

E

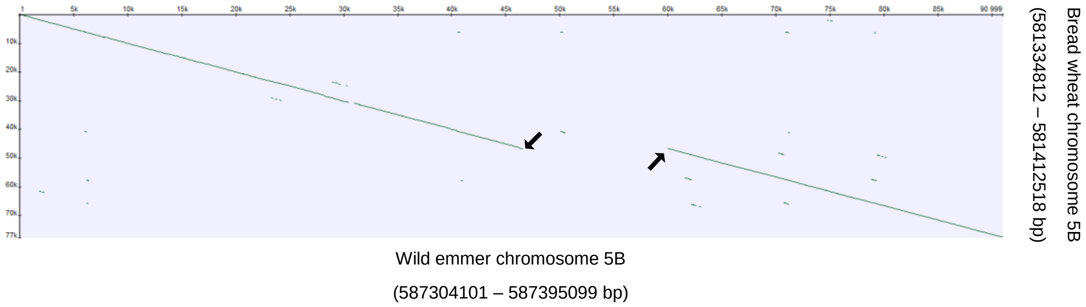
Wild emmer chromosome 5B
(587304101 – 587395099 bp)
Bread wheat chromosome 5B
(581334812 – 581412518 bp)

F

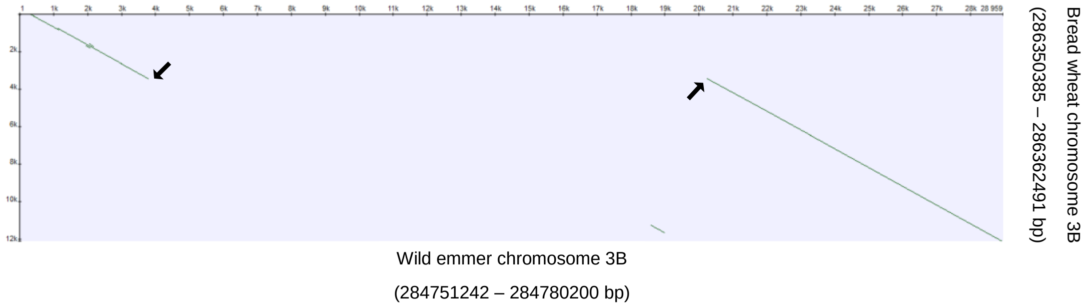
Wild emmer chromosome 3B
(284751242 – 284780200 bp)
Bread wheat chromosome 3B
(286350385 – 286362491 bp)

G

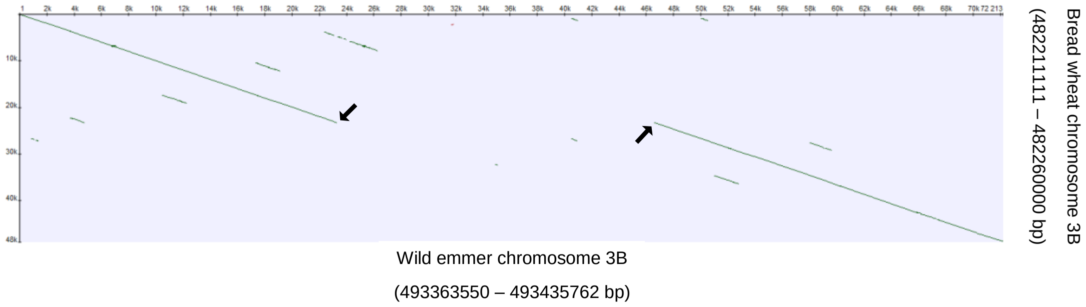
Wild emmer chromosome 3B
(493363550 – 493435762 bp)
Bread wheat chromosome 3B
(482211111 – 482260000 bp)

H

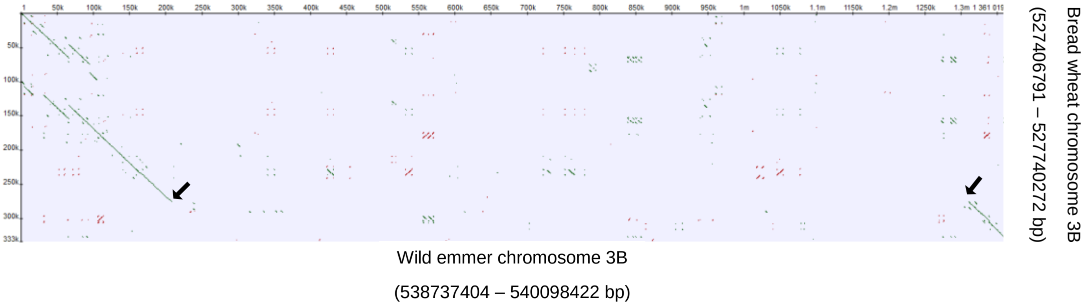
Wild emmer chromosome 3B
(538737404 – 540098422 bp)
Bread wheat chromosome 3B
(527406791 – 527740272 bp)

I

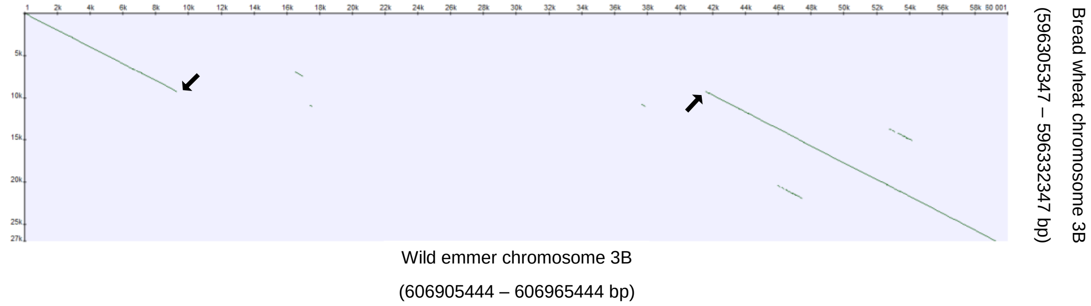
Wild emmer chromosome 3B
(606905444 – 606965444 bp)
Bread wheat chromosome 3B
(596305347 – 596332347 bp)

J

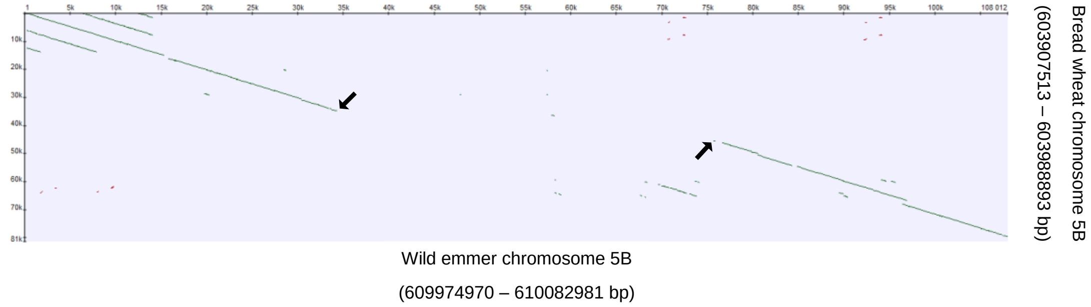
Wild emmer chromosome 5B
(609974970 – 610082981 bp)
Bread wheat chromosome 5B
(603907513 – 603988893 bp)

K

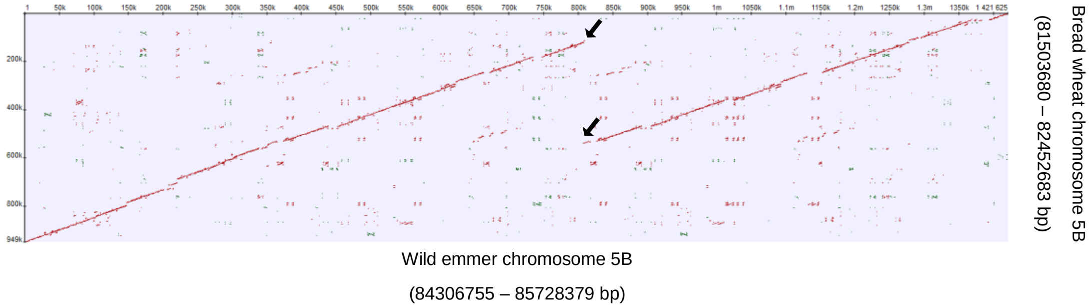
Wild emmer chromosome 5B
(84306755 – 85728379 bp)
Bread wheat chromosome 5B
(81503680 – 82452683 bp)

L

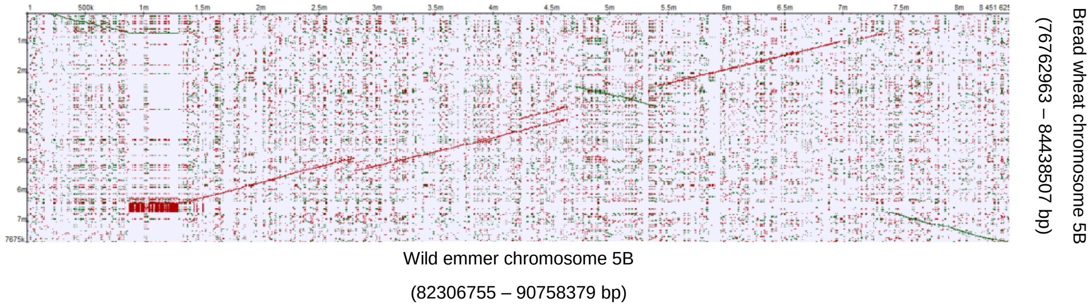
Wild emmer chromosome 5B
(82306755 – 90758379 bp)
Bread wheat chromosome 5B
(76762963 – 84438507 bp)
