# Supplementary material for: Identification and characterization of large-scale genomic rearrangements during wheat evolution
Source: PLoS One. 2020 Apr 14;15(4):e0231323. doi: 10.1371/journal.pone.0231323 (PMC7156093; doi:10.1371/journal.pone.0231323)
Supplement: S1 Table — (PDF) [file pone.0231323.s004.pdf]

**Species**	**Accession**	**Origin**
Ae. speltoides (BB)	TS47	Unknown
Ae. speltoides (BB)	542274	Turkey
Ae. speltoides (BB)	TS41	Israel
Ae. searsii (BB)	TE36	Syria
Ae. searsii (BB)	TE44	Jordan
Ae. searsii (BB)	599140	Jordan
T. turgidum ssp. *dicoccoides* (AABB)	Zavitan	Israel
T. turgidum ssp. *dicoccoides* (AABB)	TTD48	Iran
T. turgidum ssp. *dicoccoides* (AABB)	TTD20	Israel
T. turgidum ssp. *durum* (AABB)	Svevo	Italy
T. turgidum ssp. *durum* (AABB)	TTR19	Israel
T. turgidum ssp. *durum* (AABB)	TTR16	Israel
T. aestivum (AABBDD)	CS46	Unknown
T. aestivum (AABBDD)	TTA01	Unknown
T. aestivum (AABBDD)	78809	Georgia
T. aestivum (AABBDD)	377626	Former Yugoslavia
Ae. tauschii (DD)	TQ27	Israel
