# Supplementary material for: Identification and characterization of large-scale genomic rearrangements during wheat evolution
Source: PLoS One. 2020 Apr 14;15(4):e0231323. doi: 10.1371/journal.pone.0231323 (PMC7156093; doi:10.1371/journal.pone.0231323)
Supplement: S2 Table — (PDF) [file pone.0231323.s005.pdf]

Indel	Species[1]	Forward primer	Reverse primer	Annealing temperature	Product length
$5B_1$	wild emmer	GAGCCACGTAAACAAACAAGCT	ACCACGTCATCCTGGCTTTTAT	60°C	2436bp
	wild emmer	GGTACGTTTGATGACTTGTGGC	CCTAAAGGCGTTGCTATTCGTG	60°C	1345bp
	wild emmer, durum, bread wheat	GAGCCACGTAAACAAACAAGCT	GACGTCATGTTGTACCACTCCT	60°C	1998bp
$5B_3$	wild emmer, durum	GGCTTAAACCTTCTTGATCGGC	TTGTGGAGTTCTGTAGACGCAT	60°C	1361bp
	bread wheat	TTTGGTAGCGACTTGATCCGAA	TTGTGGAGTTCTGTAGACGCAT	60°C	1169bp
$3B_4$	wild emmer	ATGAACGCTCCACATCCTCC	CACTCCAAAGCTCCCGAAGA	60°C	4413bp
	durum, bread wheat	ATGAACGCTCCACATCCTCC	CGGAGTTGTGCTAGAGAGAGAG	60°C	219bp
$5B_5$	wild emmer	CTAAGTGCGGCGGTCAAATT	CTCTTCAATGGTGTTCGGCC	59°C	879bp
	wild emmer	CGCCGGTTAGTAAAAGCACC	GGGATCGGTTTGTGAGGAGA	59°C	391bp
	durum, bread wheat	TAAATTGGGCTCTCACGGGT	GGGATCGGTTTGTGAGGAGA	59°C	787bp
$5B_6$	wild emmer, durum, bread wheat	GGTTTTGCCCAACTCCGAAT	CCGGTTTGGCATCCTTTTGT	59°C	2315bp
	wild emmer	ACCCCTCAACCCTGTAAACC	CCGGTTTGGCATCCTTTTGT	59°C	2415bp

[1] Amplification was seen in the mentioned species.
