# Supplementary material for: Identification and characterization of large-scale genomic rearrangements during wheat evolution
Source: PLoS One. 2020 Apr 14;15(4):e0231323. doi: 10.1371/journal.pone.0231323 (PMC7156093; doi:10.1371/journal.pone.0231323)
Supplement: S1 Raw images — (DOCX) [file pone.0231323.s006.docx]

**Identification of large-scale genomic rearrangements during wheat evolution and the possible underlying mechanisms**

Inbar Bariah, Danielle Keidar-Friedman, and Khalil Kashkush*

Department of Life Sciences, Ben-Gurion University, Beer-Sheva 84105, Israel

^*^Corresponding author:

Khalil Kashkush

Department of Life Sciences
Ben-Gurion University
Beer-Sheva, 84105, Israel

Tel 972-8-6461206

Fax 972-8-6461276

E-MAIL [kashkush@bgu.ac.il](mailto:kashkush@bgu.ac.il)

**Running head:**

DNA rearrangements in wheat

**A**

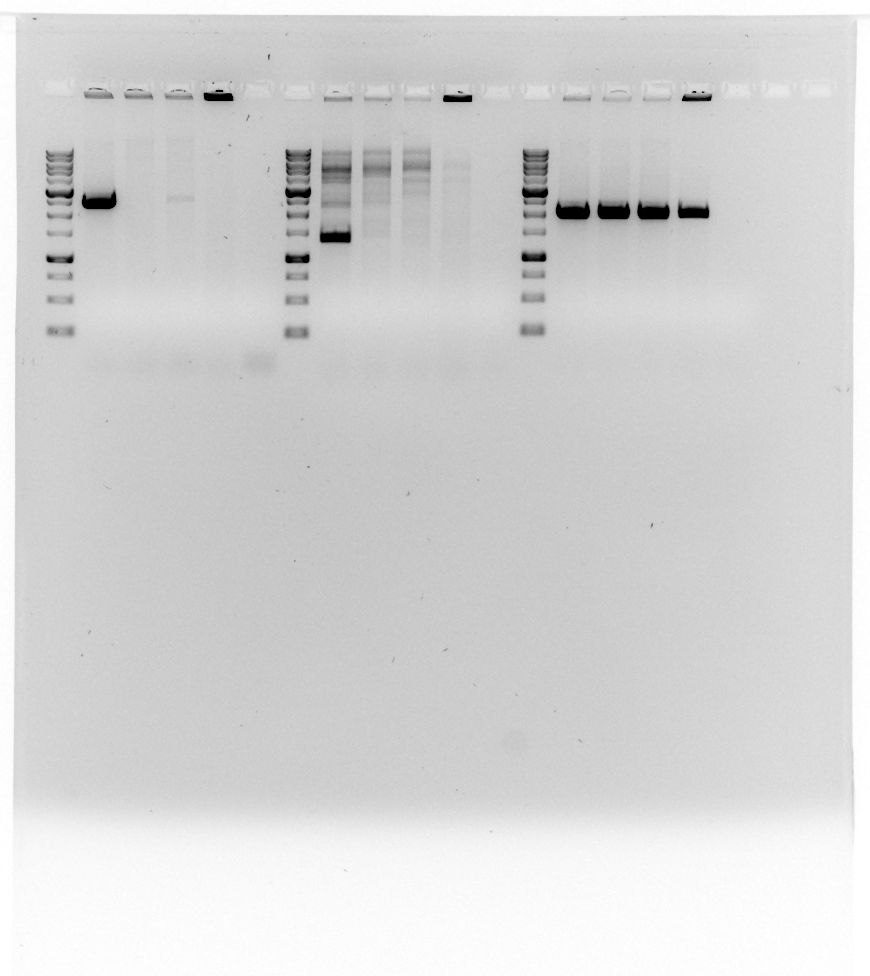


**B**

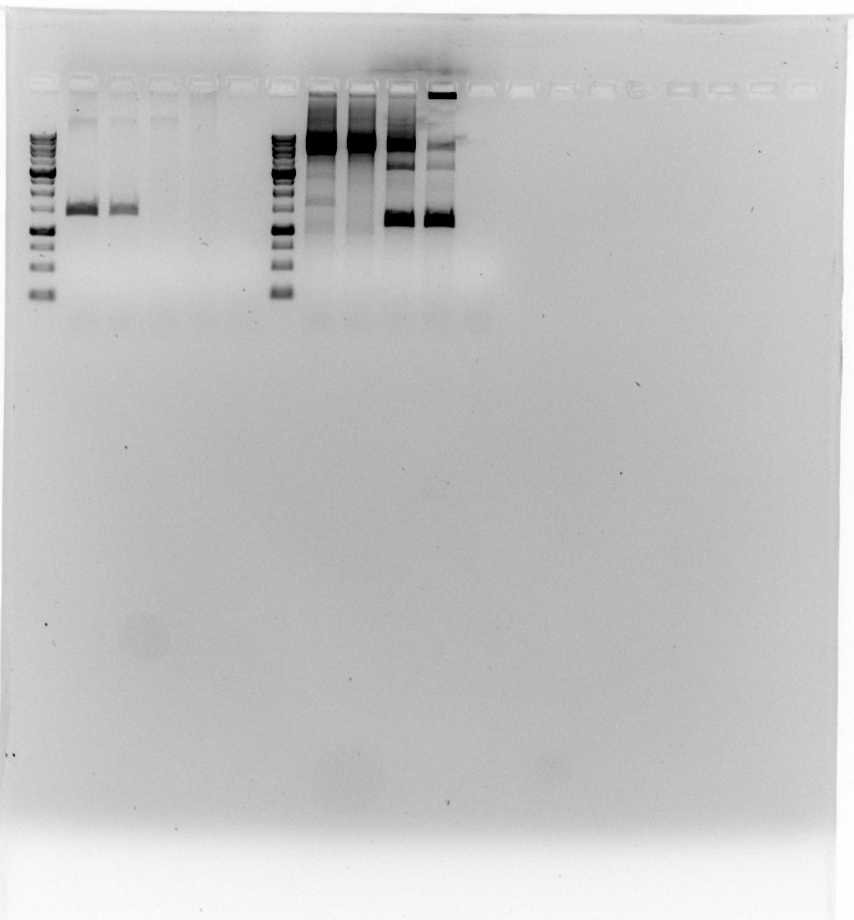


**C**

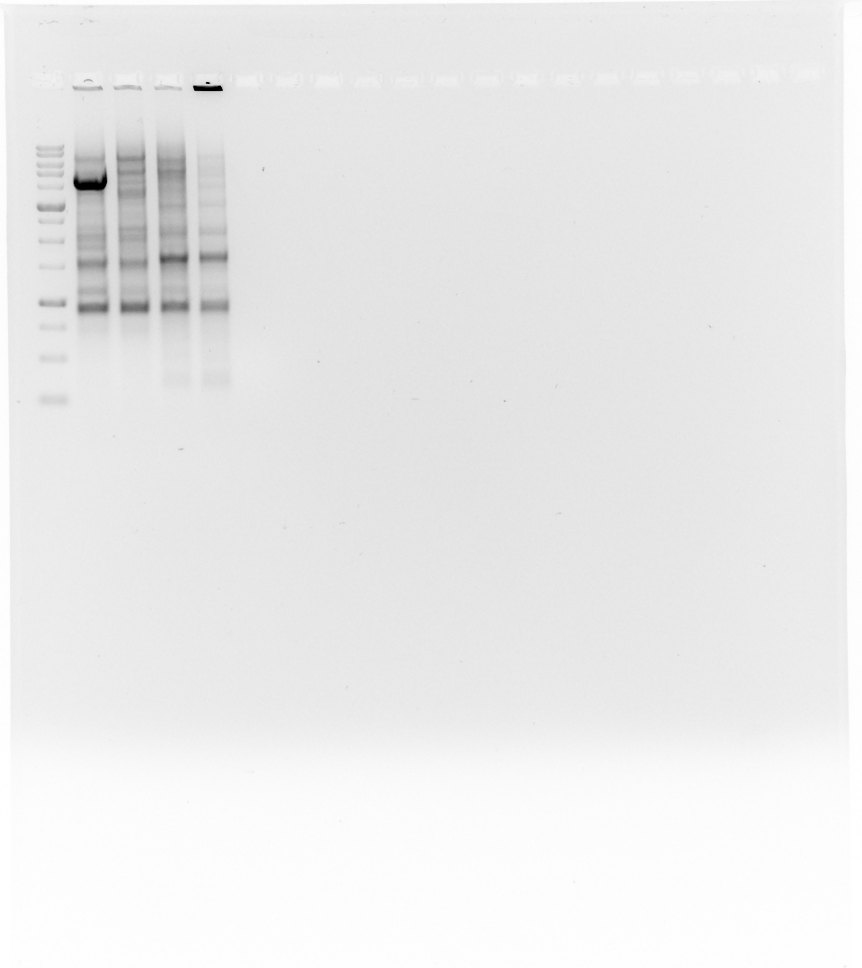


**D**

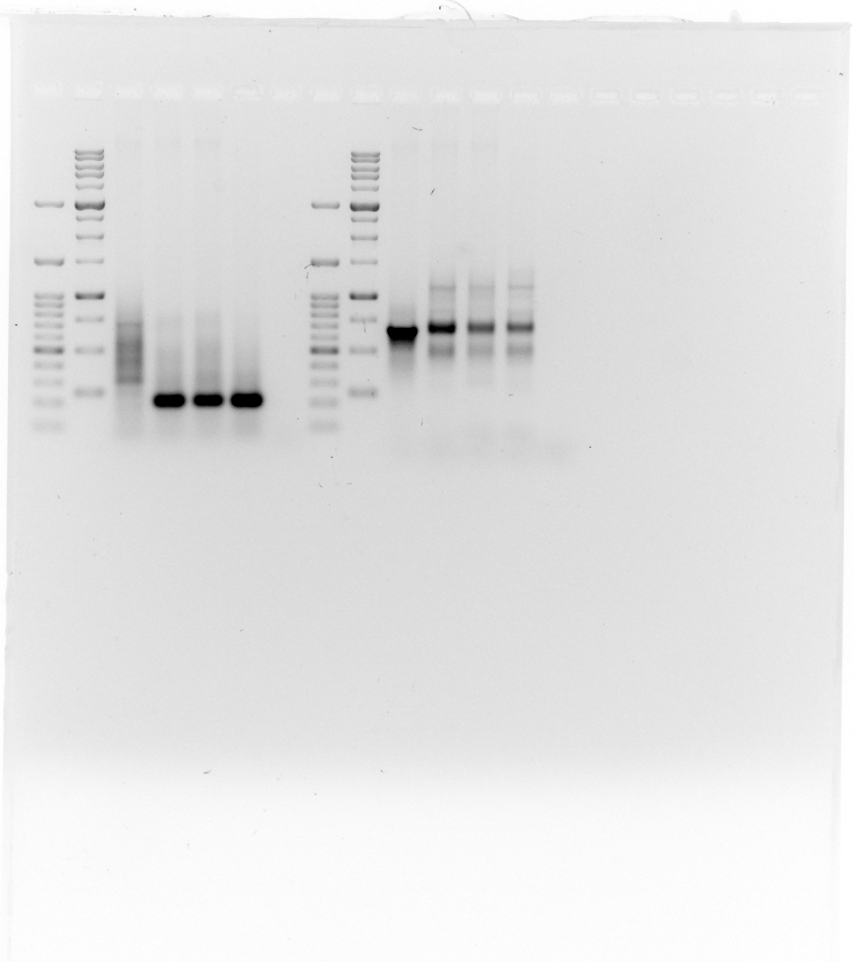


**E**

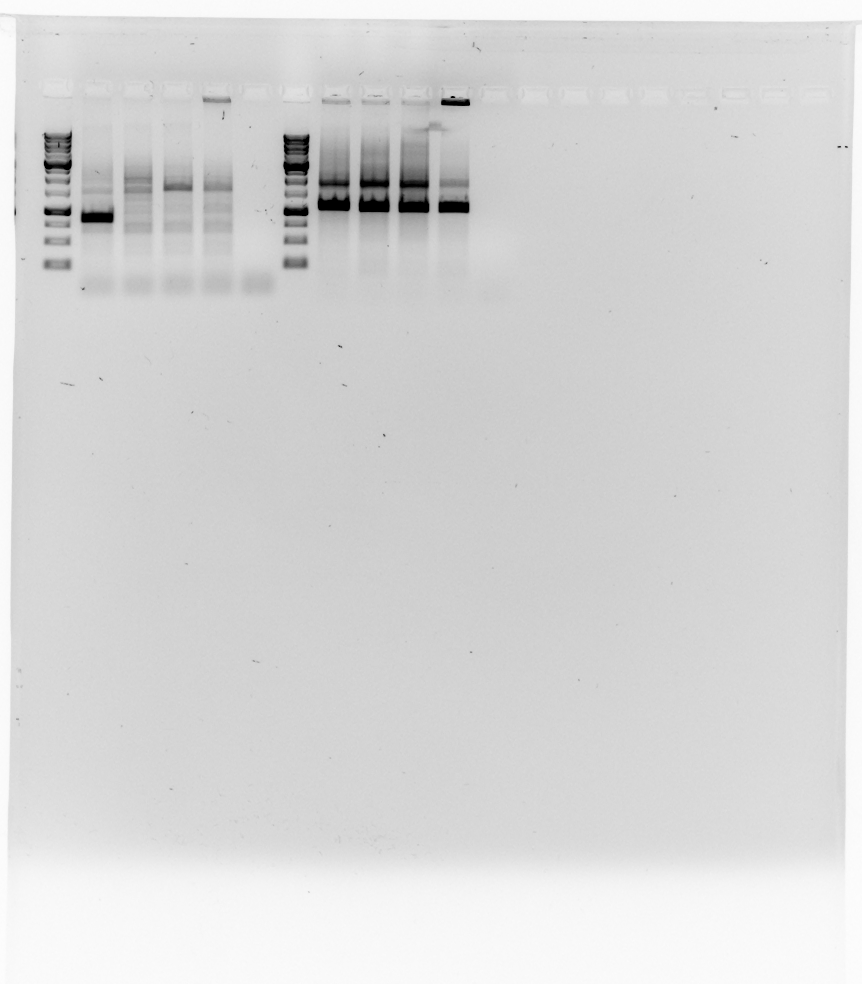


**F**


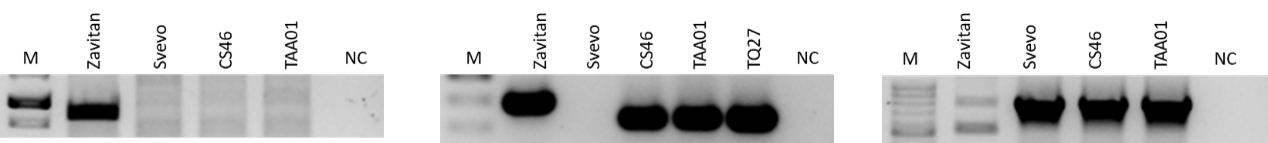


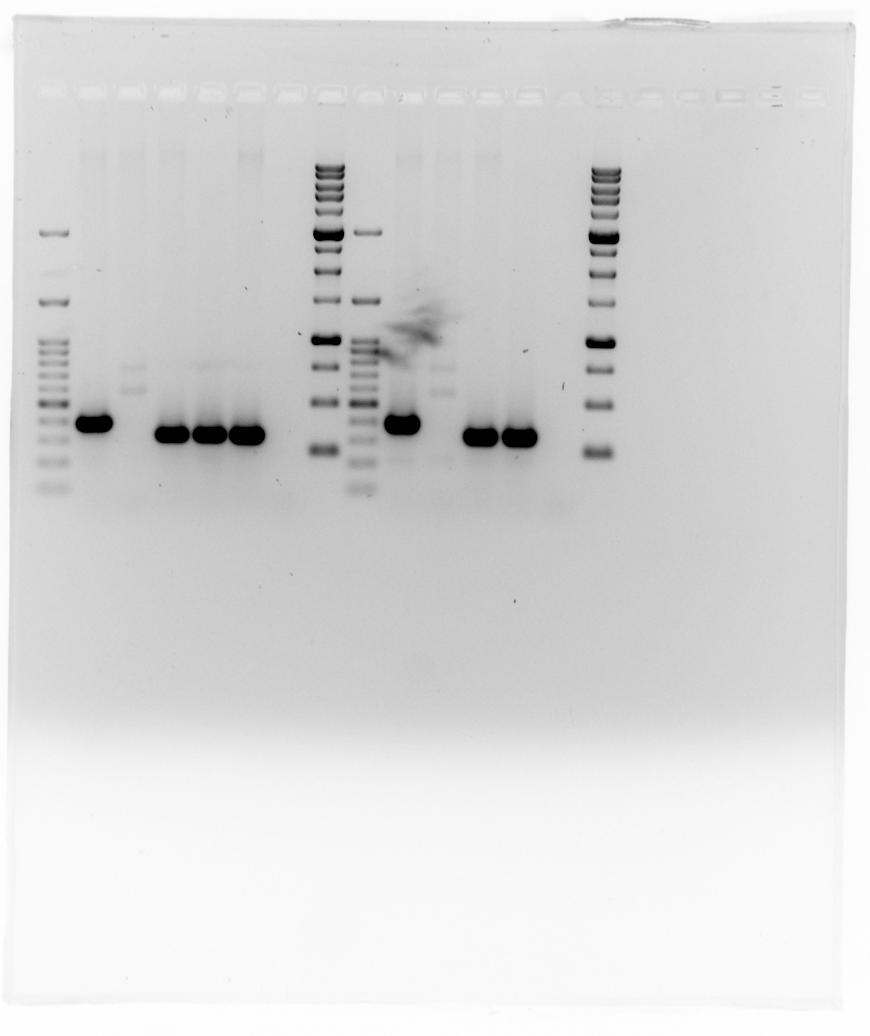


**G**

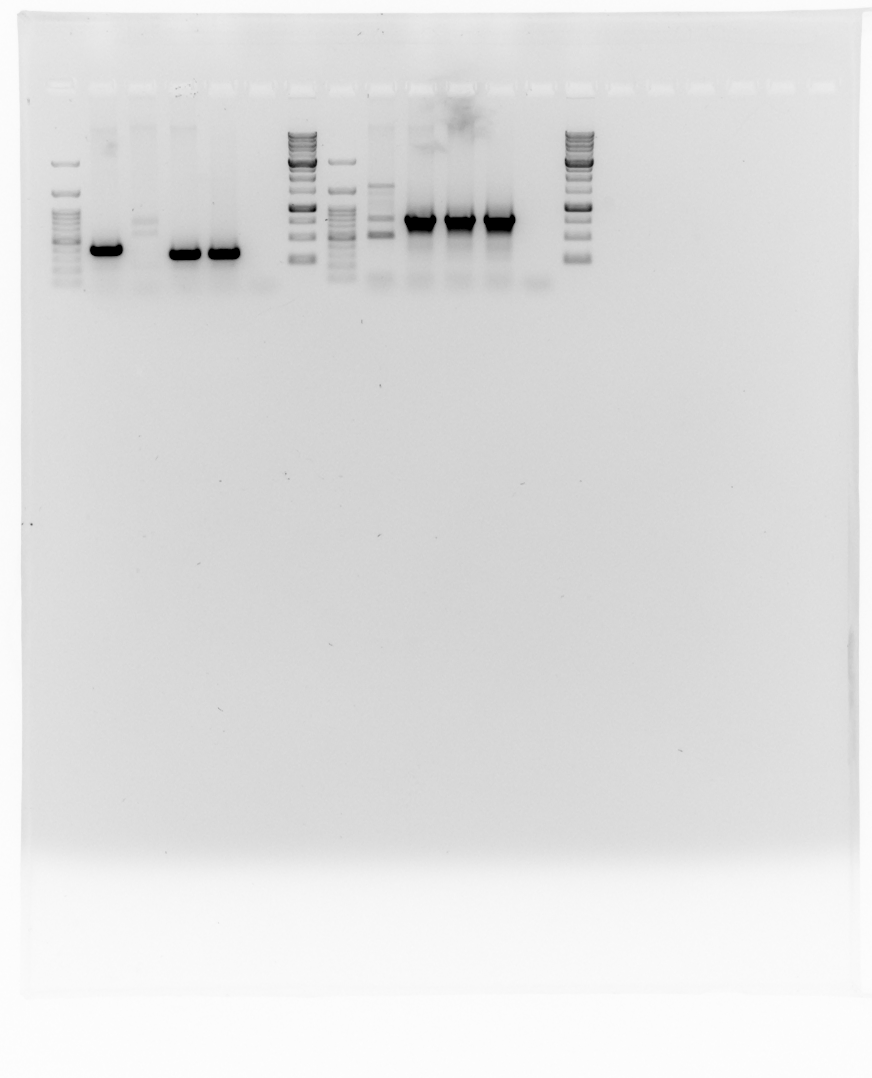


**H**

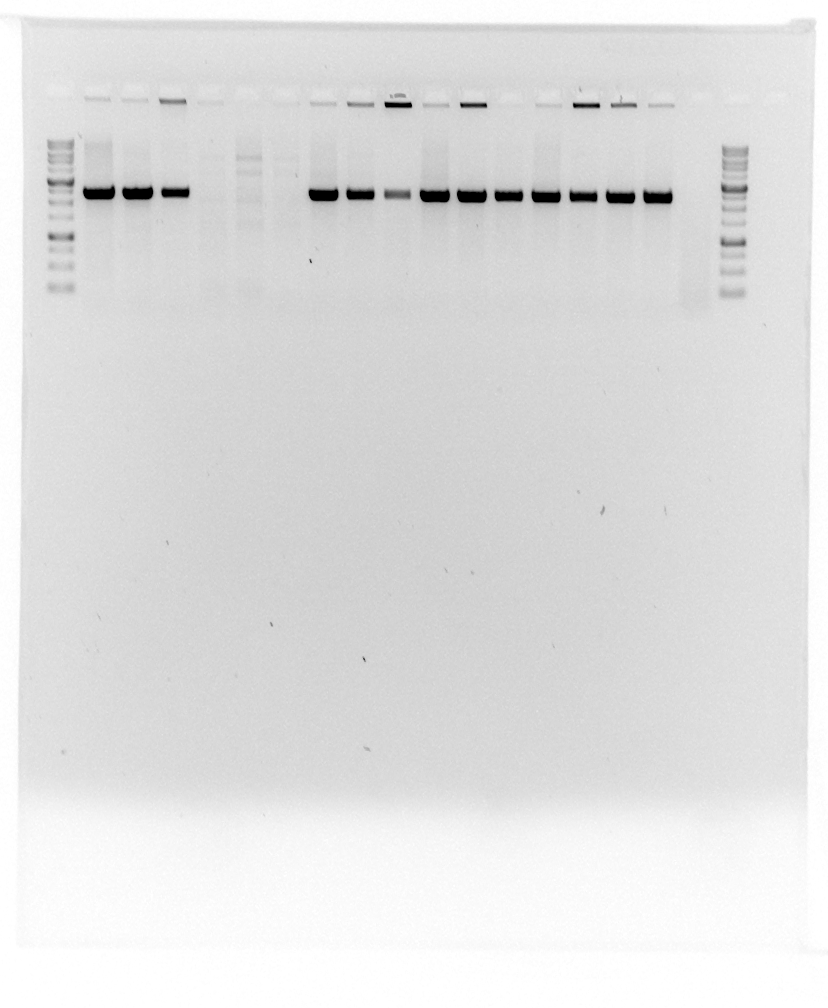


**I**

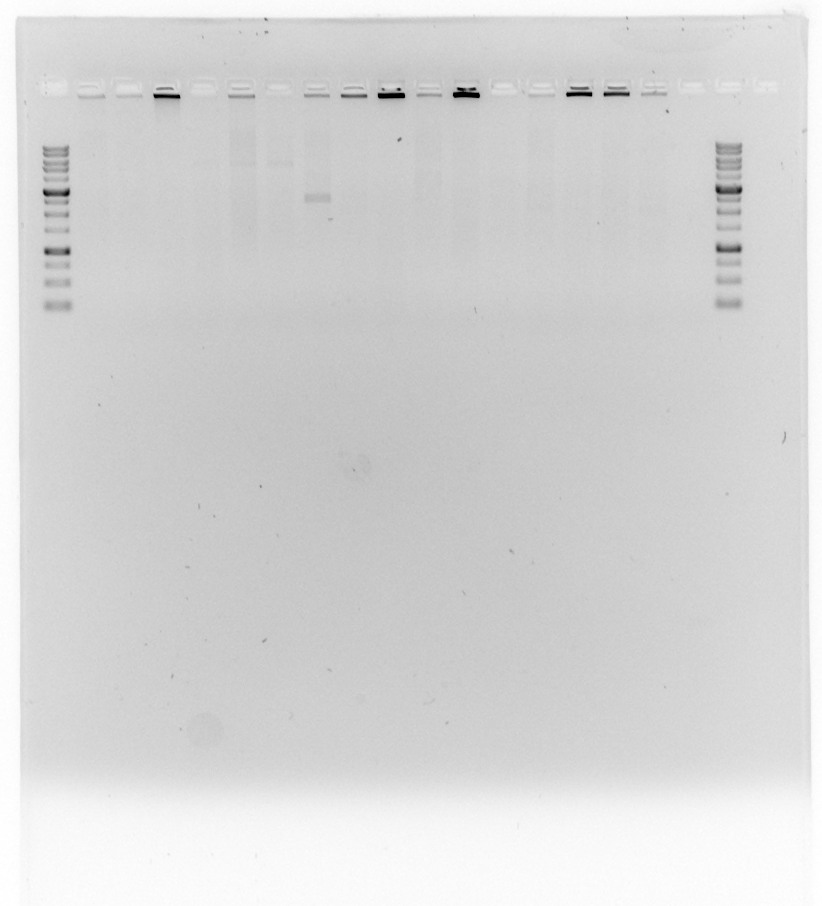


Full-length (original) gels for PCR analysis displayed in Figs 2 and 6: (A) Fig 2A-C, (B) Fig 2D-E, (C) Fig 2F, (D) Fig 2G, (E) Fig 2H, (F) Fig 2I, (G) Fig 2J, (H) Fig 6A and (I) Fig 6B. Expected products length are indicated by red arrows.

“M” represents the size marker, “NC” represents for negative control, ddH_2_0 was used as template in PCR reactions. PCR analysis were performed for 17 accessions of *Triticum* and *Aegilops* species (see S1 Table).
