# Supplementary material for: Identification and characterization of large-scale genomic rearrangements during wheat evolution
Source: PLoS One. 2020 Apr 14;15(4):e0231323. doi: 10.1371/journal.pone.0231323 (PMC7156093; doi:10.1371/journal.pone.0231323)
Supplement: Attachment — Submitted filename: Response to Reviewers comments.docx [file pone.0231323.s007.docx]

Dear Editor,

We would like to thank you and thank the reviewers for your constructive comments. Your comments allowed us to improve the data presentation and the quality of the manuscript. We have agreed to all of the comments and performed the requested changes including repeating PCR reactions for sequence validation. We provide below a point by point response to all comments. Note that page and line numbers are based on the unmarked (without track changes) version of the manuscript.

Editor comments:

1) As indicated by the AE below, the paper must be written so that the data is not summarized in context of the models. Rather than data should be more fully shown and used to derive the models. Mixing the two together is misleading and illogical. The Supplementary data are also required within the Figures section and sequences should be provided in Figures when mentioned in the text.

Response: We have made text rearrangements and separated the results and discussion sections. We also have changed the representation style of the data. Furthermore, we have included supplemental figures to the main text as suggested by the reviewers. For example; Figs. S4- 8 are now represented as Figs 2 and 6 in the text. Sequence coordinates are found in Table 1 and sequence alignments are represented as dot plots found in S2-3 Figs. Sequence comparisons and relevant annotations are found in Fig 1 and Figs. 3- 5. See below our response to reviewers’ comments.

2) The hypotheses are speculative. The speculative nature of the hypotheses need to be clearly described and should not be presented as conclusions in the title.

Response: Done. We have separated the results and discussion sections, and modified the manuscript title and some of the sections titles.

a. What was the basis of the choice of the 11 cases investigation?

Response: See text (lines 128-135). Out of the 29 *Fatima* polymorphic wild emmer-unique insertions, 20 were included within 19 loci in which *Fatima*-containing sequences were replaced by long insertions in bread wheat genome, and 9 were found within sequences that were absent from the orthologous loci in bread wheat genome. The 9 loci together with a single locus out of the 19 previously described loci, showed clear breakpoints and were chosen for further analysis. Additionally, a case where two *Fatima* insertions from the wild emmer genome showed high flanking similarity to a single *Fatima* insertion from the bread wheat genome was identified and further analyzed.

Detailed comparative analysis of the above 11 cases were done using a chromosome walking approach and dot plot sequence alignments (S2 Fig) in wild emmer vs. bread wheat genomes.

b. The indels require further sequencing to be verified.

Response: We routinely do this kind of validation for PCR products. Here as your and reviewer request, we have repeated PCR reactions, and sequenced the products and validated them by sequencing. We have mentioned this in the manuscript (in material and methods and results sections (for example see lines 619-622).

c. Place Figures S4 to S8) within the text.

Response: Done, they are now Figs 2 and 6

d. Replace accession numbers with species names in the Supplementary data 4-8.

Response: Done, see Figs 2 and 6.

e. The methods require more clarity as described by the two Reviewers. Why was MAK software used?

Response: Done. See below our response to Reviewer 2.

f. Clarify the use of Fatima as a marker for overall rearrangement.

Response: See below our response to Reviewer 2.

g. The Stacy involvement is unconvincing. Please modify to accommodate Reviewer 2's critique.

Response: Done. See below our response to Reviewer 2.

h. There are many textually vague or poorly described terms and logic (spelling, intact vs non-intact elimination versus deletion, etc, as well as evidence that Fatima activation is a late step). Please modify as suggested by Reviewers.

Response: Done. See details below

**Reviewer #1:**

1) In their paper Bariah I. and co-authors describe a list of eleven large indels associated with a transposable element (Fatima) and that segregate presence-absence variation between bread wheat and wild emmer species. The structural rearrangements are thoroughly described and followed by hypotheses about the molecular mechanisms that originated those rearrangements. Despite being explained and referred, the hypotheses remain mostly speculative, which is not always clear in the text and part of the title of the current manuscript. The eleven indels described in the manuscript were validated by PCR. However, without further sequencing of the amplified fragments, neither a molecular marker scale for the resulting electrophoresis gels (Figures S4 to S8), the validation remains unclear.

Response: The main title and the subtitles of the manuscript were changed. We also have separated the results and the discussion sections. In addition, as requested, unique PCR bands were verified by sequencing and DNA ladders were used to estimate product length (see above response to editor comment). Note that from our experience, no need for such validation to be included in the supplementary. But, if you request that, we can add Fasta format sequences. For example:

>5B5_zavitan_expected_391bp_product

CGCCGGTTAGTAAAAGCACCTGTTTCTCTCAAAAAAAAAAGTAAAAGCACCTGTAACTGACGCTCAGGGCGCCAAATAGGATTCGCCCGCACCCGCGCCCGCACCGCAACAGCATCCAGGTCGCCAGGTCGGTCCTCTAACCGGAGTCAAACTCAACCACGCGCTCCCCTCTGCGCCGAACCCTCCTCCCCCTTTCGCCAGAACACCCCGGAAGCATCTGGAAGCCCGGCCCTCAGCCCCCGCCACCGCCCGATCCGGATCCGACGGTCCGCGCCCGCCTCTCCCCCGCCCTCCCGCATCTTCGAGAGCGTTCACTCCCGACCACCCGTCCCGCGCTCCGCTATAACTACGACCGCCCCCTCTCCCCCACCTCTCCTCACAAACCGATCCC

>Forward_primer_5B5_zavitan_391bp_product

TGTTYYAAAAAAARTAAAAGCACCTGTAACTGACGCTCAGGGCGCCAAATAGGATTCGCCCGCACCCGSGCCCGCACCGMAACAGCATCCAGGTCGCCAGGTCGGYCCTYTAACCGGAGTCAAACTCAACCACSCSCTCCCCTYTGSGCCGAACCCTCCTCCCCCTTTCGCCARAACACCCCGGAAGCATYKGGAAGCCCGGCCCTMAGCCCCCGCCACCGCCCGATCCGGATCCRACGGTCCGSGCCCGCYTYTCCCCCGCCYTCCCGMATYTTCRARAGCGTTMACTCCCGACCACCCGTCCCGSGCTCCGYTWTAACTACRACCGCCCCCTYTCCCCCACCTYTCCTMACAAACCGATCCMA

>Reverse_primer_5B5_zavitan_391bp_product

TTGTGGGGGGGGCGGTCGTAGTTATAGCGGRCGCGGGACGGGTGGTCGGGAGTGAACGCTCTCGAAGATGCGGGAGGGCGGGGGAGAGGCGGGCGCGGACCGTCGGATCCGGATCGGGCGGTGGCGGGGGCTGAGGGCCGGGCTTCCAGATGCTTCCGGGGTGTTCTGGCGAAAGGGGGAGGAGGGTTCGGCGCAGAGGGGAGCGCGTGGTTGAGTTTGACTCCGGTTAGAGGACCGACCTGGCGACCTGGATGCTGTTGCGGTGCGGGCGCGGGTGCGGGCGAATCCTATTTGGCGCCCTGAGCGTCAGTTACAGGTGCTTTTACTTTTTTTTTTRARARAAMCAGGKGYTTTWMYAAMCCCGGSGA

2) The main figures (figure 1 to 4) simplify well the long description of the given rearrangements but I think the “real” alignments would fit in the supplementary data as well or a figure supporting the “high nucleotide identity” at those sequences.

Response: Sequence coordinates are found in Table 1 and the genome assemblies used here are publicly available. Sequence alignments are represented as dot plots found in S2-3 Figs. Since some of the loci are over 1 Mb in length, we avoided full sequence alignments. Sequence comparisons and relevant annotations are found in Figs 1 and 3-5.

3) In the text, to which subfigure (A-B-C-D) it refers is missing.

Response: Fixed

4) In figures S4 to S8 I would mention the species and not the accession for clarity

Response: Done, they are now Figs 2 and 6

5) In methods, the genome assemblies and annotation (gene and TE) used in the study are unclear, I would refer to the accession numbers.

Response: Done under " Wheat genomic data" and "Identification and characterization of Fatima-containing sequences that undergo indel and of indel breakpoints".

**Reviewer #2:**

1) The authors conducted comparative analyses of Triticum aestivum (bread wheat) and Triticum turgidum ssp. Dicoccoides (wild emmer) Fatima transposable elements and flanking sequences. The authors described some results showing that some insertions/deletions (indels) occurred via unequal intra-strand recombination or double-strand break (DSB) events, and that a number of these events occurred at or near Fatima and other TEs.
   The main conclusion of the paper is that massive large-scale DNA rearrangements induced by transposons played a prominent role in wheat speciation. The authors show that a number of rearrangement breakpoints are located within Fatima transposons; however, this result is expected based on the method by which these cases were selected for study. In addition, other breakpoints are located in non-Fatima TEs. Thus it is difficult from the data presented to assess the true proportion of TE-induced rearrangements, and whether this figure is greater than would be expected by random chance considering that 80% of the wheat genome is composed of transposon sequences.

Response: In this work, *Fatima* were used as a genetic marker here and was chosen since it was showed to underwent elimination in the first four generations of a newly formed wheat allohexaploid. It was not claimed that *Fatima* elements were involved in the mechanisms leading for the 11 indels. TEs might have a role in the rearrangements, i.e., as sequence repeats enabling unequal intra-strand recombination. For clarification, the line discussing the TEs abundant in indels break points was removed from the abstract and the roll of *Fatima* as genetic marker was further discussed in the text.

2) Line 100, the authors stated that “The consensus sequence of the autonomous Fatima element was used as a query”. Additional description of the query sequence is given in Materials and Methods, but some of this information should be presented in the paper main text (the length of the complete element and LTRs).

Response: Done (lines 97-98)

3)The authors should indicate what criteria are used to distinguish “intact” from “non-intact” elements.

Response: The terminology was corrected, the retrieved elements are not necessarily intact, but full-length elements (line 101)

4) Finally, it would be of some interest to indicate the number of solo LTRs derived from full-length Fatima.

Response: Since *Fatima* was used as a genetic marker, *Fatima* content in the genome was not the focus of this study. *Fatima* content and distribution in the bread wheat genome was recently described in a paper [1] (see references).

5) Line 101, “MAK software designed to retrieve Fatima insertions”, the authors should explain why this software is used in this study. Based on the information from the software website, MAK stands for MITE Analysis Kit, which is designed to facilitate automated analysis of miniature inverted repeat transposable elements (MITEs). While Fatima is a retrotransposon, the authors should describe the reason that using this MITE tool to analysis a retrotransposon.

Response: Since *Fatima* was used as a genetic marker, it was efficient to retrieve *Fatima* insertions base one sequence similarity. MAK enables the retrieval of TEs based on sequence similarity and specifically similarity of the terminal regions of the sequence. Clarification is added to the text (line 99). For this reason, the retrieved elements were defined as a specific variant of *Fatima* and no general claims were made regarding the content and distribution of *Fatima* in wheat genomes.

6) Line 114, Indicate why these particular chromosomes were chosen for analysis? Why is the location of Ph1 on 5B important to this study?

Response: Changed (see lines 114-115).

7) Line 123-129. Authors should replace percentages (“5%, 57%, 34% and 4% of the cases”) with actual numbers.

Response: Done

8) Line 134: How were these 11 cases chosen for study?

Response: see our response above to editor comment. Explained in lines 128-135.

9) Line 147: Authors state that “Fatima was most likely activated following allohexaploidization”; the reasoning here is a little murky. Possibly could be helped by summarizing number of copies eliminated. Also, is there any evidence to show that the transposon was inactive before the allohexaploidization?

Response: It can be deduced from the fact that 80 insertions (~15% of the insertions analyzed here) were found to be wild emmer specific and yet the total number of insertions was similar in wild emmer and bread wheat. Activation fallowing allohexaploidization was previously shown for *Fatima* as mentioned in lines 80-82.

10) Line 251: DBS, should be DSB

Response: Done

11) Line 347: templet; should be template?

Response: Done

12) Line 411, 412: regarding the proposed introgression of an 11 kb segment: Can the TEs contained within this segment indicate its probable origin?

Response: detail analysis using TREP and in NCBI did not provide any strong hits. We also compered to wild emmer and *Ae. tauschii* with BLAST+ stand-alone version 2.2.24 and did not find any hit.

13) Lines 467-469: The potential role of Stasy elements in the segmental duplication is overstated. The first Stasy is 2.5 kb from the duplication endpoint, and the second is indicated as “in the 5’ region” of a 470 kb duplication. As depicted in Figure 4, this second Stasy may be 50 kb or more from the duplication endpoint. If these elements were involved in the duplication events, one would expect them to be present at or very close to the breakpoints.

Response: We have mentioned that sequence length is unscaled in the Figs. The presence of a truncated *Stasy* element (SINE) 2.5 kb downstream of the first repeat start in wild emmer and of a highly similar (99%) truncated *Stasy* element 2.5 kb downstream of the second repeat. Based on *Stasy* location, we speculated that *Stasy* might be involved (see lines 404-406).

14) Line 772: bends, should be bands

Response: Done

15) Line 775: revers, should be reverse

Response: Done

16) In this paper, do ‘elimination’ and ‘deletion’ carry the same meaning?

Response: indeed. And we agree with you, thus we have corrected to “deletion”.

17) Some loci described in the study have “high” TE content (Lines 186, 214, 230, 263). Considering that wheat genome is already 80% TE sequences, what does “high” mean?

Response: The TE content is only stated as part of the sequence annotation and characterization, and was not claimed to be high. For clarification, the line discussing the TEs abundant in indels break points was removed from the abstract.

18) It is recommended that Supplemental Figure 7 be included in the manuscript as a main text figure. In fact, Supplemental Figures 4, 5, 6, and 7 could all be combined into a single figure for the main text. These PCR results don’t take much space, and it would be helpful for these data to be readily available to the reader.

Response: Done. They are now Figs 2 and 6.
