# Supplementary material for: Identification and characterization of large-scale genomic rearrangements during wheat evolution
Source: PLoS One. 2020 Apr 14;15(4):e0231323. doi: 10.1371/journal.pone.0231323 (PMC7156093; doi:10.1371/journal.pone.0231323)
Supplement: Attachment — Submitted filename: Response letter.docx [file pone.0231323.s008.docx]

Dear editor,

We want to thank you for the constructive comments, and especially we want to thank reviewer #1 for his very critical reading of the manuscript. We have addressed all of your minor comments (see below our point by point response to all comments).

Response to editor comments:

1. There is a need for further explanation of the logic for using the MITE method to map the Fatima elements.

Response: MITE analysis kit (MAK) software (<http://labs.csb.utoronto.ca/yang/MAK/>) is a homology-based software, meaning it uses a consensus sequence as query and BLASTN algorithm with global alignment. This software was originally developed by Guojun Yang, University of Toronto (Janicki et al. 2011; Yang and Hall 2003a) and devoted for retrieving MITE insertion, so it was named accordingly. However, the MAK can be used for any given query sequence in order to be retrieved from genome draft. We previously used MAK to retrieve retroelements such as *Au*-SINE [see our previous publications: Ben-David, S., Yaakov, B ., and Kashkush, K. (2013). Genome-wide analysis of short interspersed nuclear elements (SINEs) revealed high sequence conservation, gene association and retrotranspositional activity in wheat. *The Plant Journal*. 76(2): 201-210; Danielle Keidar, Chen Doron, and Khalil Kashkush (2018). Genome-wide analysis of a recently active retrotransposon, Au SINE, in wheat: content, distribution within subgenomes and chromosomes, and gene associations. *Plant Cell Reports*. 37(2):193-208]. The big advantage of using the MAK software is that it is very efficient and allows the retrieving of flanking sequences. We have stated the fact that MAK can be used for any TE sequence in lines 290-292.

2. Please incorporate each of the experimental, explanatory, and textual comments of Reviewer 1 into the text.

Response: See our below response to the reviewer’s comments

3. The labels in Fig 2 are difficult to read. Please use a different font or size.

Response: Done. See corrected figure 2.

4. Please provide lane designations and other labels for Figure 4.

Response: Done. We have corrected the legends of all figures, and included all details. See figure legends.

5. It clearer representation of the correlation of the raw data to the data presented in Figure 2 and Figure 4 is required. Please provide a simple statement in the raw data document of the correspondence of the raw data to the data in the Figures.

Response: Done. The raw data refers to figures 2 and 6. Please see the corrected legend in the bottom of “S1_raw_images”.

Response to Reviewer’s comments:

l.11 > might have occurred.

Response: Done.
l.54 > rephrase to “shaped the wheat genome”?

Response: Done.
l.68 > as (was) reported.

Response: Done.
l.77 > duplicated above (between wheat allopolyploids).

Response: Previous paragraph refers to newly formed wheat allopolyploids, while here we refer to wild relatives and different cultivars. Different articles were brought as references to support the different statements.

l.91 > structural rearrangements have no “role” in domestication per se, but rather the selection involved during the domestication process will shape the patterns of rearrangements we observe?

Response: we agree with the reviewer and we have erased this sentence.

l.98 > still unclear how/why MAK was specifically designed to retrieve Fatima insertions. Isn’t the tool designed for MITE elements (inverted repeats)? How is it different from a blast search? or other available tools designed for LTRs?

Response: MITE analysis kit (MAK) software (<http://labs.csb.utoronto.ca/yang/MAK/>) is a homology-based software, meaning it uses a consensus sequence as query and BLASTN algorithm with global alignment. This software was originally developed by Guojun Yang, University of Toronto (Janicki et al. 2011; Yang and Hall 2003a) and devoted for retrieving MITE insertion, so it was named accordingly. However, the MAK can be used for any given query sequence in order to be retrieved from genome draft. We previously used MAK to retrieve retroelements such as Au-SINE [see out previous publications: Ben-David, S., Yaakov, B ., and Kashkush, K. (2013). Genome-wide analysis of short interspersed nuclear elements (SINEs) revealed high sequence conservation, gene association and retrotranspositional activity in wheat. *The Plant Journal*. 76(2): 201-210; Danielle Keidar, Chen Doron, and Khalil Kashkush (2018). Genome-wide analysis of a recently active retrotransposon, Au SINE, in wheat: content, distribution within subgenomes and chromosomes, and gene associations. *Plant Cell Reports*. 37(2):193-208]. The big advantage of using the MAK software is that it is very efficient and allows the retrieving of flanking sequences. We have stated the fact that MAK can be used for any TE sequence in lines 290-292.

l.105 > unmapped wording confusing

Response: corrected to “unassigned to a specific chromosome”.

l.107+113 > reasons confusing to me > rephrase focusing on the fact that the B sub-genome is more variable and bears important genes?

Response: corrected. We have erased this statement.
l.109 > remove “(termed the pivotal genome)“

Response: Done.
l.118 > Comparative analysis add a note “see Methods part Identification of species-specific Fatima insertions”.

Response: Done.
l.125 > unclear how this would happen, wasn’t the assemblies used to map the indels and not reads?

Response: The word “reads” was removed. The insertions might be monomorphic, however, due to Ns in the assembly they were identified as wild emmer unique previous to manual analysis.

l.128-130 > I would clearly state why you’re focusing on those 9 “species specific” insertions.

Response: “The 9 loci together with a single locus out of the 19 previously described loci, showed clear breakpoints and **thus** were chosen for further analysis.”
l.138 > “no sequence similarity” to low sequence similarity?

Response: Done.
l.157 > “some nucleotide identity“ to sequence homology? Also small paragraph that can be included with the following one?

Response: We refer to single nucleotide identity in some of the cases. However, we changed this to “sequence homology” as was suggested by the reviewer. The small paragraph refers to 9 different cases, while the following refers only to 3 cases described under the subtitle “Indels flanked by long sequence repeats”. The other 6 cases are discussed under the subtitle “Indels flanked by short sequence repeats”.

l.160 > absent sequences to sequences absent.

Response: Done.
l.172 > coding for a lipoxygenase.

Response: Done
l.176 to 180 > methods.

Response: Done
l.341-342 > duplicate of l.333-334.

Response: Done
l.345 > unclear why “via recurrent backcrossing” here. Also I would tone down the introgression statement as there is no strong evidence for it. ?

Response: the definition “via recurrent backcrossing” was adapted from [1].

The statements regarding introgression are rephrased based on the reviewer

suggestion.
l.360 > “in the sequence coverage” a bit misleading? Rather alignment?

Response: Done
l.379 > source to origin?

Response: Done
l.380 > transpired to emerged?

Response: Done
l.509 and further > complimentary to complementary

Response: Done
l.537 > acting via to promoting?

Response: Done
